# Supplementary material for: Interspecific Hybridization between the Two Sympatric Termite Reticulitermes Species under Laboratory Conditions
Source: Insects. 2019 Dec 23;11(1):14. doi: 10.3390/insects11010014 (PMC7022586; doi:10.3390/insects11010014)
Supplement: Supplementary file 1 [file insects-11-00014-s001.zip › insects-654640-Suplementary/insects-654640-supplementary-resubmit.pdf]

Supplementary Materials for

Interspecific hybridization between the two sympatric termite *Reticulitermes* species under laboratory conditions

Jia Wu, Huan Xu, Ali Hassan, Qiuying Huang*

Hubei Insect Resources Utilization and Sustainable Pest Management Key Laboratory, Huazhong Agricultural University, Wuhan 430070, Hubei, China.

*Corresponding author: Qiuying Huang

E-mail: qyhuang2006@mail.hzau.edu.cn

Supplementary materials for this manuscript include the following:

Table S1

The termite sample information used in this study

Table S2

Primer sequences of the five microsatellite loci used in the present study

Table S3

Genotypes of parents and larvae from the five colonies at the five microsatellite loci

Movie

Mating behavior observed in the lab

Table S1 The termite sample information used in this study*.

Items	Colonies	Combination	replicates	Time of collecting
Aggression	A_1, A_2, A_3 A_4, A_5 B_1, B_2, B_3, B_4, B_5	A_1B_1, A_2B_2, A_3B_3, A_4B_4, A_5B_5	*30*	Late Mar. 2018
Tandem	A_1, A_2, A_3 A_4, A_5 B_1, B_2, B_3, B_4, B_5	A_1B_1, A_2B_2, A_3B_3, A_4B_4, A_5B_5	*30*	Late Mar. 2018
Allogrooming	A_1, A_2, A_3 A_4, A_5 B_1, B_2, B_3, B_4, B_5	A_1B_1, A_2B_2, A_3B_3, A_4B_4, A_5B_5	*30*	Late Mar. 2018
Mating	A_1, A_2, A_3 A_4, B_1, B_2, B_3, B_4,	A_1B_1, A_2B_2, A_3B_3, A_4B_4,	*8*	Late Mar. 2018
Genotyping analyses	A_1, A_2, A_3 A_4, A_5 B_1, B_2, B_3, B_4, B_5	A_1B_1, A_2B_2, A_3B_3, A_4B_4, A_5B_5	*5*	Late Mar. 2018

*Species A: *R. flaviceps*; Species B: *R. chinensis*.

Table S2 Primer sequences of the five microsatellite loci used in this study*.

Locus	Primer sequence 5′(r) 3′	Size (bp)	Ta (°C)	Repeat unit
Ra141	F: CACATTTGAGGTTCGCAAGA	168	56	$(TTA)_8$
	R: GCCAGAAGGCCAATTACAGA			
Ra 144	F: CAAATAGAGCTCCGTGTTTCG	158	55	$(TTAG)_7$
	R: CCATAGAAACCTCCGAAAGG			
Rs03	F: TCCTGACTGTACAAAGAAAAGTGG	230	56	$(CT)_9$
	R: TGGCATCAAGCTACGTATTCA			
Rs76	F: AATCCGGGGAATTTCTTGAC	187	58	$(AGTT)_8$
	R: CTGCATAACGATGTCTGCGT			
Rs78	F: GCTTCTCAAGAAGGACTGTGC	178	58	$(AGTT)_7$
	R: GCCCCAGTTGAGATATGGAA			

*Repeat units and sizes refer to the sequenced alleles. F, forward; R, reverse; Ta, annealing temperature (°C).

Table S3 Genotypes of parents and larvae from the five colonies at the five microsatellite loci*.

Colony ID	individual	SSR Loci*				
		*Rs*78	*Rs*76	*Rs*03	*Ra*141	*Ra*144
A1B1-4	RC♀	177/188	169/174	238/238	188/203	155/159
	RC♂	172/188	174/187	232/238	201/103	155/159
	RF♀	164/177	162/182	232/238	188/201	155/155
	RC♂	188/188	169/174	238/238	195/195	155/159
	L1	164/188	162/174	238/238	188/195	155/155
	L2	172/177	162/174	238/238	201/103	155/159
	L3	**172/188**	162/174	232/238	**203/203**	155/159
	L4	164/188	162/174	238/238	201/203	155/155
	L5	**164/172**	**162/187**	232/238	**195/201**	159/159
	L6	188/188	**162/187**	--/--	201/103	155/155
	L7	164/188	162/174	232/238	188/195	155/159
	L8	164/188	174/174	232/238	188/195	155/155
	L9	164/188	174/174	238/238	**188/201**	155/155
	L10	164/188	**174/187**	238/238	188/195	159/159
	L11	172/188	**162/187**	--/--	188/195	155/155
	L12	172/188	162/174	238/238	188/195	155/159
	L13	172/177	**162/187**	232/238	195/195	155/155
	L14	**172/188**	**174/187**	238/238	188/195	159/159
	L15	172/177	**174/187**	232/238	188/195	155/155
	L16	**172/188**	**174/187**	238/238	188/195	155/159
A2B2-2	RC♀	164/172	162/174	232/238	188/195	155/155
	RC♂	177/181	169/174	232/232	195/197	155/155
	RF♀	164/172	178/182	232/238	195/195	155/159
	RC♂	172/181	169/174	238/238	188/201	155/159
	L1	164/172	162/174	232/232	188/195	155/155
	L2	172/177	174/174	238/238	195/201	155/159
	L3	164/172	174/174	238/238	195/201	155/155
	L4	172/177	174/182	232/232	**188/201**	155/159
	L5	172/177	174/174	238/238	188/195	**159/159**
	L6	172/177	162/174	238/238	188/195	**159/159**
	L7	172/181	174/178	232/232	195/201	155/155
	L8	164/172	162/174	232/232	188/195	155/159
	L9	172/177	--/--	232/232	195/201	**159/159**
	L10	172/177	162/174	238/238	195/201	155/159
	L11	172/181	--/--	232/232	188/195	**159/159**
	L12	172/181	169/174	238/238	188/195	**159/159**
	L13	172/181	169/174	232/232	188/195	155/159
	L14	172/177	162/174	238/238	--/--	155/155
	L15	164/172	162/174	238/238	**188/201**	155/159
	L16	172/181	162/174	238/238	**188/201**	155/155

	L17	172/177	162/174	238/238	**188/201**	155/159
	L18	172/177	174/178	232/232	--/--	**159/159**
	L19	172/177	174/178	232/232	188/195	**159/159**
	L20	164/172	174/178	238/238	188/195	155/155
A3B3-1	RC♀	164/172	174/174	238/238	188/195	155/163
	RC♂	164/177	169/174	238/238	195/195	155/155
	RF♀	164/177	162/174	232/232	188/188	159/159
	RC♂	164/172	174/187	232/238	188/195	--/--
	L1	164/177	162/174	238/238	188/195	155/163
	L2	164/164	**162/169**	232/238	188/195	155/155
	L3	164/177	**162/278**	238/238	188/195	155/163
	L4	**177/177**	162/174	238/238	195/195	159/159
	L5	164/164	169/174	232/238	188/188	155/155
	L6	172/177	**162/187**	232/238	188/195	159/159
	L7	164/164	174/174	238/238	188/195	155/155
	L8	164/177	174/174	238/238	188/195	159/159
	L9	164/177	174/174	**232/232**	188/195	155/155
	L10	**177/177**	174/174	238/238	195/195	159/159
	L11	164/172	174/174	232/238	188/188	159/159
	L12	172/177	174/174	238/238	188/195	155/163
	L13	--/--	174/174	**232/232**	188/195	155/155
	L14	164/177	174/174	238/238	188/188	155/155
A4B4-2	RC♀	177/177	174/174	232/232	195/201	155/159
	RC♂	164/177	162/174	232/238	195/195	155/159
	RF♀	164/172	162/174	238/238	188/201	155/155
	RC♂	177/181	174/174	232/238	195/195	155/159
	L1	164/177	**162/162**	238/238	188/195	155/155
	L2	**164/181**	174/174	238/238	188/195	155/159
	L3	164/164	162/174	232/232	188/195	155/159
	L4	**164/181**	174/174	232/238	195/201	155/155
	L5	164/177	174/174	238/238	195/201	159/159
	L6	164/177	**162/162**	238/238	195/195	155/155
	L7	**164/181**	174/174	232/238	195/201	155/159
	L8	**164/172**	174/174	238/238	195/195	155/155
	L9	**164/181**	162/174	232/232	195/201	155/155
	L10	**177/181**	174/174	238/238	195/201	159/159
	L11	**177/181**	174/174	232/238	195/195	155/155
	L12	177/177	174/174	238/238	195/201	155/155
A5B5-6	RC♀	165/177	171/183	232/242	193/202	--/--
	RC♂	165/177	171/183	232/238	190/205	155/155
	RF♀	177/177	167/175	232/232	190/190	159/172
	RC♂	177/177	175/175	232/232	190/193	159/172
	L1	177/177	**175/183**	232/232	**193/202**	155/159
	L2	177/177	**175/175**	232/232	190/193	159/159

L3	177/177	**167/175**	232/232	190/190	159/159
L4	177/177	**175/175**	232/232	190/193	159/159
L5	165/177	**167/175**	232/238	190/193	--/--
L6	177/177	**167/175**	232/232	190/193	159/159
L7	177/177	**167/175**	232/232	190/193	159/159
L8	177/177	**167/175**	232/232	190/190	159/159
L9	177/177	**171/175**	232/232	190/190	155/159
L10	177/177	**175/175**	232/232	190/190	159/159
L11	177/177	**175/175**	232/232	190/190	159/159
L12	177/177	**171/175**	232/232	**193/193**	155/159
L13	165/177	**175/183**	232/242	**193/193**	--/--
L14	165/177	**171/175**	232/242	190/193	155/159
L15	177/177	**167/175**	232/232	190/190	159/159
L16	177/177	**175/175**	232/232	190/193	159/159
L17	165/177	**171/175**	232/232	190/193	--/--
L18	177/177	**167/175**	232/232	190/190	159/159

* Hybrid diagnosed loci were shown in red color. Pure-breed diagnosed loci were shown in blue color. Uncertain diagnosed loci were shown in black color. --/-- shows that the loci of this individual samples failed to PCR.
